# A multimodal magnetoencephalography 7 T fMRI and 7 T proton MR spectroscopy study in first episode psychosis

**DOI:** 10.1038/s41537-020-00113-4

**Published:** 2020-09-04

**Authors:** Timothy J. Gawne, Gregory J. Overbeek, Jeffery F. Killen, Meredith A. Reid, Nina V. Kraguljac, Thomas S. Denney, Charles A. Ellis, Adrienne C. Lahti

**Affiliations:** 1 Department of Optometry and Vision Science, University of Alabama at Birmingham, Henry Peters Building, 1716 University Blvd, Birmingham, AL 35233 USA; 2 Department of Psychiatry and Behavioral Neurobiology, University of Alabama at Birmingham, Sparks Center 560, 1720 7th Ave. S., Birmingham, AL 35233 USA; 3 Health Science Foundation Neurology, University of Alabama at Birmingham, Sparks Center Suite 350, 1720 7th Ave. S., Birmingham, AL 35233 USA; 4 Department of Electrical and Computer Engineering, Auburn University, 3101 Shelby Center, Auburn, AL 36849 USA; 5 Wallace H. Coulter Department of Biomedical Engineering, Georgia Institute of Technology and Emory University, 313 Ferst Dr. Rm 2127, Atlanta, GA 30332 USA

**Keywords:** Schizophrenia, Psychosis

## Abstract

We combined magnetoencephalography (MEG), 7 T proton magnetic resonance spectroscopy (MRS), and 7 T fMRI during performance of a task in a group of 23 first episode psychosis (FEP) patients and 26 matched healthy controls (HC). We recorded both the auditory evoked response to 40 Hz tone clicks and the resting state in MEG. Neurometabolite levels were obtained from the anterior cingulate cortex (ACC). The fMRI BOLD response was obtained during the Stroop inhibitory control task. FEP showed a significant increase in resting state low frequency theta activity (*p* < 0.05; Cohen *d* = 0.69), but no significant difference in the 40 Hz auditory evoked response compared to HC. An across-groups whole brain analysis of the fMRI BOLD response identified eight regions that were significantly activated during task performance (*p* < 0.01, FDR-corrected); the mean signal extracted from those regions was significantly different between the groups (*p* = 0.0006; *d* = 1.19). In the combined FEP and HC group, there was a significant correlation between the BOLD signal during task performance and MEG resting state low frequency activity (*p* < 0.05). In FEP, we report significant alteration in resting state low frequency MEG activity, but no alterations in auditory evoked gamma band response, suggesting that the former is a more robust biomarker of early psychosis. There were no correlations between gamma oscillations and GABA levels in either HC or FEP. Finally, in this study, each of the three imaging modalities differentiated FEP from HC; fMRI with good and MEG and MRS with moderate effect size.

## Introduction

Schizophrenia is associated with impairments in cognitive control, a neuropsychological process thought to contribute to a wide range of cognitive deficits in schizophrenia^1^. There is strong evidence that schizophrenia is an extremely heterogenous disorder^2^, quite likely involving multiple more-or-less independent pathophysiological mechanisms^3^. Studies using only a single brain imaging modality would of necessity be limited in determining if they are quantifying different aspects/expressions of a single underlying pathophysiological mechanism, or are instead quantifying aspects/expressions of a mixture of different pathophysiological mechanisms. Therefore using diverse brain imaging techniques on the same subject population might be especially valuable in the study of schizophrenia.

Both functional MRI (fMRI) and magnetoencephalography (MEG) allow the measurement of brain function; these are complementary modalities that, respectively, provide high spatial and temporal resolution. While fMRI measures hemodynamic changes that generate the blood oxygen level-dependent (BOLD) signal, MEG allows the examination of fast neural oscillations, which enable coordinated brain activity fundamental to cognition. Studies of MEG/ electroencephalography (EEG) during a resting state in schizophrenia show considerable variability of results, but one of the more consistent observations is of an increase in low-frequency spontaneous brain activity (Delta and Theta bands, 1–4 and 4–8 Hz, respectively) in patients with schizophrenia (SZ) than healthy controls (HC)^4,5^. Low frequency oscillations are thought to support synchronization between distant brain regions^6,7^. This is important as functional connectivity between remote brain regions measured with fMRI has been shown to be altered in schizophrenia^8^.

Higher frequency cortical activity in the gamma band (nominally 30–80 Hz, although there is no widely accepted formal definition) have also been suggested to be critical for brain function, especially for local cortical processing^6^. However, it can be very hard to accurately record cortical gamma-band activity from the scalp surface^9^. An exception is the response of auditory cortex to 40 Hz tones, which uniquely results in cortical evoked responses that can be clearly and robustly detected at the scalp surface^10^. The activity of auditory cortex literally resonates at the same 40 Hz rate as the auditory stimulus, and this signal can be easily picked up by both MEG and, with somewhat greater difficulty, EEG. This is sometimes referred to as the “Auditory Steady State Response,” or ASSR, because during the 40 Hz stimulation there is a “steady state” of one click every 25 ms. In schizophrenia, including in first episode psychosis (FEP), deficits in various aspects of the evoked response to audio tone bursts in the high frequency (Gamma) range (40 Hz) have been identified in numerous studies^11–21^.

Theoretical and experimental evidence implicate post-synaptic currents as a common origin of both the fMRI and MEG signals, suggesting that while these signals have different properties, they share a common underlying electrophysiological process^22^. In addition, γ-aminobutyric acid (GABA) interneurons, especially the fast-spiking ones that express the Ca-binding protein parvalbumin (PV), play a fundamental role in the control of the synchrony of cortical pyramidal neurons by producing rhythmic inhibitory postsynaptic potentials. These interneurons appear to be key to the generation of gamma oscillations^23^. For example, optogenetic manipulations of PV interneurons that reduce or increase their activity can either suppress or amplify gamma oscillations, respectively^24^. Thus, GABA inhibition controlling postsynaptic currents is likely to participate in the shaping of MEG signals. In healthy controls, combined MEG/MR spectroscopy (MRS) studies investigating the relationships between gamma oscillations and GABA levels in the visual^25,26^ and motor^27^ cortex have demonstrated the presence^26,27^ or the absence^25^ of such correlations. Likewise, MEG/MRS studies using a pharmacological challenge with GABA-modulating drugs, such as diazepam^28^, tiagabine^29^, and propofol^30^, to evaluate whether changes in GABA levels affect gamma oscillations have also provided conflicting results.

In schizophrenia, postmortem studies have consistently identified a reduction in the expression of GAD67, the enzyme responsible for GABA synthesis, in PV interneurons^31^. GABA alterations in postmortem studies have been identified in multiple brain regions, including the prefrontal cortex (PFC), anterior cingulate cortex (ACC), hippocampus, temporal cortex, including the primary auditory cortex^31^. In addition, a number of recent MRS studies have revealed abnormal GABA measurements in schizophrenia^32,33^. These findings therefore raise the question whether, in schizophrenia, abnormal gamma oscillations are related to altered GABA levels. In a small combined EEG/MRS study, a significant correlation was observed between gamma oscillation and dorsolateral PFC GABA levels in the combined group of patients with schizophrenia and controls, both at rest and across working memory stages^34^.

There have are three previously published manuscripts based on this data set. One used MRS spectroscopy to evaluate the levels of neurometabolites independently of any other modality^35^. Another investigated the relationship between cortical glutamate/GABA and task related fMRI BOLD networks^36^. A third examined the relationships between the whole-cortex patterns of resting state functional connectivity as determined by both fMRI and MEG^37^.

In this multimodal study, we combined MEG, fMRI, and MRS to evaluate the relationship between neural oscillations and both BOLD and GABA measurements in a group of FEP patients and matched HC, allowing us to study some of the neural correlates of psychosis without confounds of illness chronicity and long term antipsychotic medication exposure. High-frequency activity can potentially have better detectability with MEG compared to EEG, especially for auditory cortex which lies in the wall of a sulcus and is thus much better aligned to generate a MEG than an EEG signal^38,39^. Because higher field strength magnets provide a better signal-to-noise ratio and spectral resolution, we used a 7 T magnet to measure GABA from a dorsal ACC voxel, as well as the BOLD response during a cognitive control task known to activate a neural circuit that includes the dorsal ACC. The ACC, a core region of the cognitive control network^40^, consistently demonstrates altered functional activation during cognition in schizophrenia^41–43^. With MEG, we obtained oscillatory measurements during a resting state, and in addition recorded the auditory evoked response (AER) to audio tones delivered at 40 Hz. In principle, this allows us to probe the ability of the cortical circuitry to generate and maintain gamma-band oscillations.

1. We hypothesize that we would replicate findings of an increase in low frequency oscillations during the resting state, as well as alterations in gamma oscillations with auditory evoked responses.
2. In addition, based on the discussed literature, we hypothesize that gamma frequency oscillations would correlate with GABA measurements in healthy controls, but that correlation would be impaired in FEP.

We additionally conducted an exploratory analysis to determine if alterations in low frequency oscillations in FEP compared to HC would correlate with alterations in the task-related BOLD response. Finally, this study will allow for the first time for the comparison of the relative effect sizes of several neuroimaging modalities that individually have been previously shown to separate FEP from HC.

## Results

### MEG resting state

**Fig. 1:**
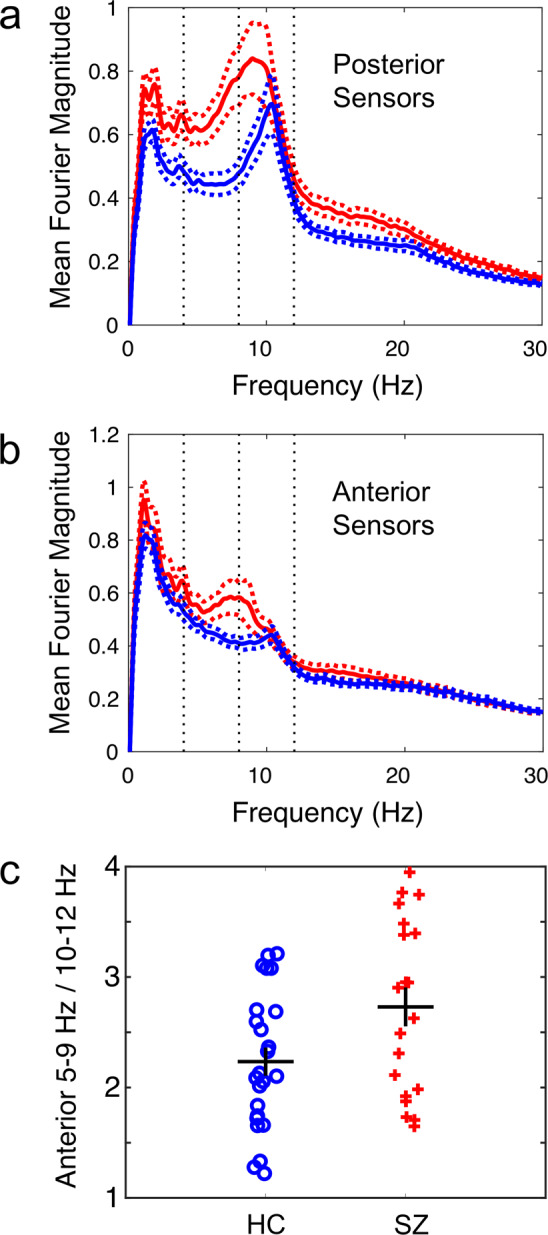
MEG resting state. **a** Mean magnitude of the Fourier transform (arbitrary units) across the posterior MEG sensors during the resting state (eyes closed). Red line is the mean of the FEP (*n* = 20, mean ± stderr), blue line is the mean of the HC (*n* = 24). **b** Same as in **a** only for the anterior sensors. **c** Ratio of the area under the Fourier transform curve from 5 to 9 Hz (high theta/low alpha) over the area from 10 to 12 Hz (high alpha) for each individual subject. HC were different from FEP by *t*-test *p* = 0.025.

At the magnetometer sensor level, the mean of the absolute value of the Fourier transform for the posterior sensors is shown in (Fig. 1a): as expected, the HC show a large peak in the mid/high alpha band. The FEP, however, on average showed considerable activity in the high-theta/low-alpha band. (Fig. 1b) is arranged in a similar manner only for the anterior sensors. Again, as expected, the HC alpha activity is considerably reduced away from the occipital lobe, but the elevated high theta/low alpha seen in the FEP persists.

There appears to be a slight increase in beta band activity for FEP vs. HC (range about 12–20 Hz), but it seems likely that this is simply a harmonic from the low alpha/theta activity (if the lower frequency oscillations are not completely sinusoidal, they will produce activity at frequencies that are integer multiples of the fundamental frequency)^44^.

Figure 1c shows the ratio of the area under the Fourier transform curve from 5 to 9 Hz (theta/low alpha range) vs. from 10 to 12 Hz (mid/high alpha range) for each individual subject. We use a ratio because the absolute magnitude of the Fourier spectrum varied from subject to subject. There was overlap between the groups, but 7 FEP had higher values of this metric than the highest HC. HC were different from FEP by *t*-test, *p* = 0.025, *t* = −2.3295, df = 42. This metric was not significant for the whole head or posterior sensors alone: while theta was larger in absolute magnitude for both posterior sensors or whole head for the FEP, the amount of theta for HC was both low and less variable in the anterior region for HC, thus increasing the divergence between the groups.

### MEG auditory evoked potentials

**Fig. 2:**
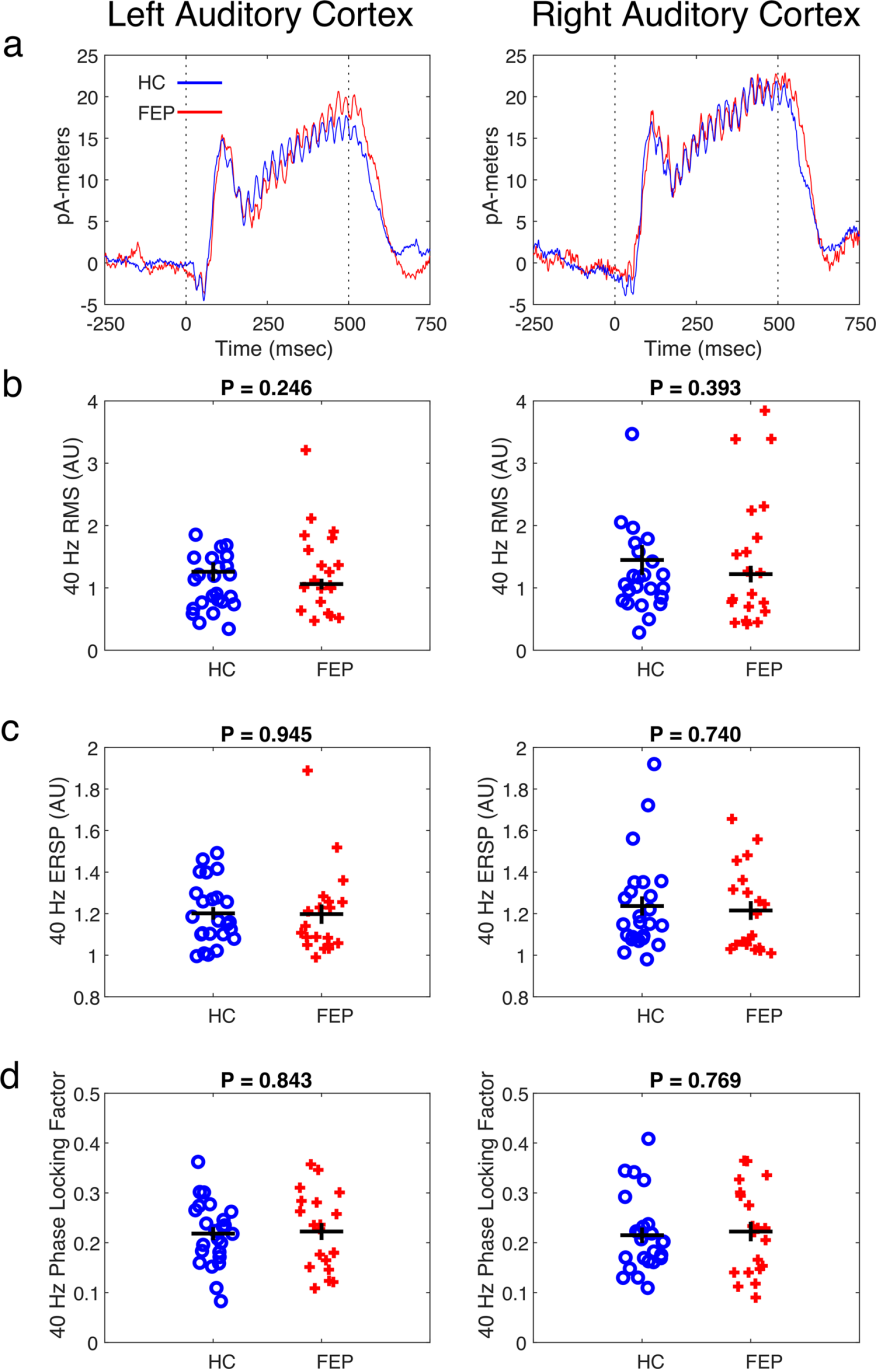
Results of the MEG auditory evoked response to 500-ms duration 40 Hz tone bursts. **a** Mean amplitude as a function of time for both FEP (red lines) and HC (blue lines) for both left and right auditory cortices. The time at which the audio stimuli began and ended are indicated by vertical dashed lines. The mean time courses of the curves for HC and FEP are similar. **b** RMS power at 40 Hz for the evoked (time-averaged phase-locked to stimulus onset) responses. Each red cross represents the mean value for one FEP, and each blue circle represents the mean value for one HC. There was not a significant difference between the HC or FEP by *t*-test (*p*-values given about each plot). **c**, **d** Plots arranged as in **b**, only for the event-related spectral perturbation (ERSP) and the phase locking factor.

Figure 2a shows the time course of the pre-attentive MEG auditory evoked responses for both HC and FEP, for both left and right auditory cortices (averaged across all trials, time-locked to stimulus onset). As expected, after a delay, there was an abrupt onset of MEG activity, and strong entrainment to the 40 Hz tones. After the tones stopped, the MEG responses stopped after a delay. Figure 2b shows the root mean square (RMS) power of the evoked response, averaged time-locked to stimulus onset, and bandpass-filtered between 35 and 45 Hz, for each individual subject. There was no significant difference between HC and FEP by *t*-test (Left cortex: *p* = 0.246, *t* = −1.177, df = 42; Right cortex: *p* = 0.393, *t* = −0.862, df = 42). Figure 2c shows the event related spectral perturbation (ERSP), a measure that captured the total increase in power at 40 Hz relative to baseline, and counts both that aspect of the response that is time-locked to stimulus onset, and that aspect of the response that is not time locked. We found no significant difference by *t*-test between FEP and HC (Left cortex: *p* = 0.945, *t* = 0.069, df = 42; Right cortex: *p* = 0.740, *t* = 0.33, df = 42). Figure 2d shows the result of calculating the Phase Locking Factor (or Intertrial Coherence), and again, there was no significant difference between FEP and HC for this metric by *t*-test (Left cortex: *p* = 0.843, *t* = −0.199, df = 42; right cortex: *p* = 0.769, *t* = −0.295, df = 42).

### MRS spectroscopy

In the same participants, we have previously reported that glutamate and total *N*-acetyl aspartate (tNAA), but not glutamine or GABA, were significantly lower in FEP patients than in HCs^45^. The results are replotted in Supplementary Fig. 1 for convenience.

### fMRI BOLD stroop

**Fig. 3:**
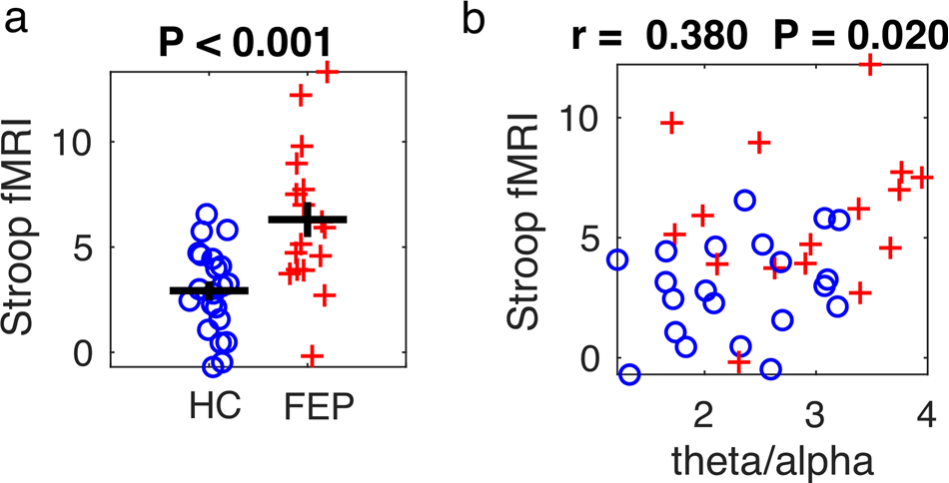
Stroop fMRI BOLD response and correlation with resting state theta/alpha ratio. **a** Plot of the Stroop effect fMRI signal incongruent > congruent for both HC (blue circles) and FEP (red crosses) for the mean of all eight regions (lower right sub-panel of Supplementary Fig. 2). **b** Correlation of the mean of all eight regions that showed a significant Stroop effect from panel a vs. the resting state theta/alpha ratio (Fig. 1c). Correlation coefficients and significance levels are given above the subplots.

An across-groups whole-brain analysis of the Stroop fMRI BOLD response to Incongruent trials minus Congruent trials was performed (*p* < 0.01, FDR-corrected)^36^. Eight regions that each significantly differentiated the Stroop fMRI signal Incongruent > Congruent trials were identified (Supplementary Table 1). The value of these eight signals, and the mean of all eight signals, is plotted for HC and FEP in Supplementary Fig. 2. Five of the eight regions separated HC from FEP by *t*-test, *p* < 0.05, although all eight regions showed a clear trend for FEP > HC. As illustrated in (Fig. 3a), the mean of the value of all eight regions also significantly differentiated FEP from SZ via *t*-test, *p* < 0.001, *t* = −3.775, df = 36 (full statistics of the eight individual regions given in Supplementary Fig. 2). Thus, it seems that the same basic pattern of the FEP having a larger fMRI Stroop effect than HC is relatively broadly distributed across the cortex.

### Exploratory analysis: correlations MEG/BOLD

The resting state theta/alpha ratio was the only aspect of the MEG results that significantly separated FEP from HC. Therefore, as shown in (Fig. 3b), we explored the correlation between the resting state MEG theta/alpha ratio and the fMRI BOLD Stroop effect. We found that across all subjects the theta/alpha ratio correlated significantly with the fMRI BOLD Stroop effect, *p* = 0.020, df = 35 (mean of all activated regions). However, the correlation between these variables was not significant when considered for HC or FEP separately.

### Correlations MEG/MRS GABA

**Fig. 4:**
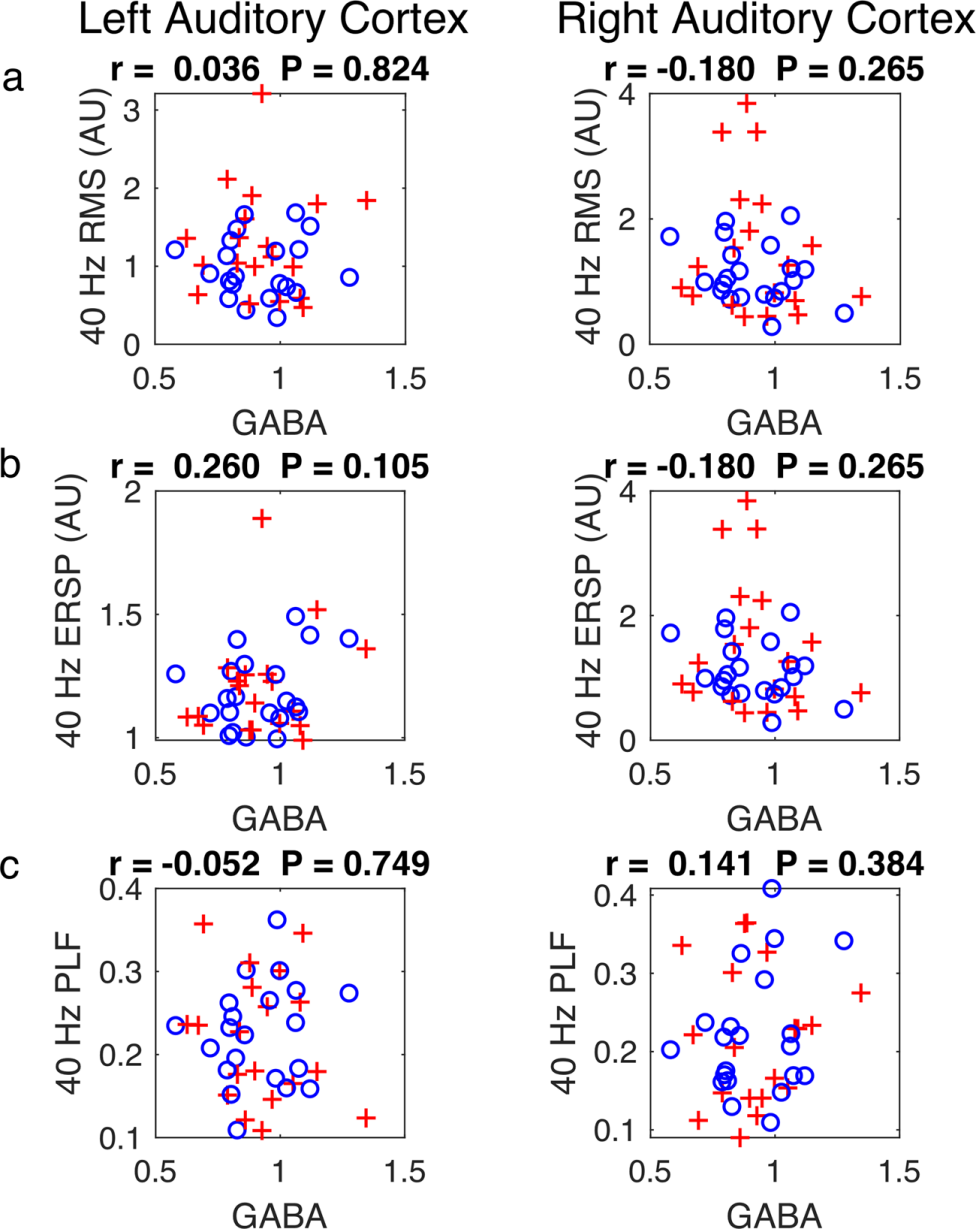
Correlations of ACC MRS GABA with the three metrics for the 40 Hz auditory steady state response (see Fig. 2) for both left and right auditory cortices. MRS GABA concentration in the ACC is shown in Supplementary Fig. 1. df = 40 for all correlations. **a** RMS root mean square power of the raw signal. **b** ERSP event-related spectral perturbation. **c** PLF phase locking factor.

As explained in the introduction, there is considerable evidence implicating the activities of GABA-ergic interneurons with gamma-band cortical activity. However, there were no significant correlations between the three metrics of the auditory response to 40 Hz tones and GABA levels (Fig. 4). An analysis looking for such correlations for the FEP and HC subjects considered separately was also negative.

## Discussion

To our knowledge, this is the first combined 7 T MRS/fMRI and MEG and the first to investigate the relationship between cortical auditory evoked gamma oscillations and GABA levels in FEP patients. The main findings are as follows: 1) FEP showed a significant increase in resting state low frequency theta activity, but no significant difference in the 40 Hz auditory evoked response compared to HC; 2) Both in FEP and HC, there was a lack of correlation between the 40 Hz auditory evoked response and GABA levels; 3) In the combined FEP and HC group, there was a significant relationship between the BOLD signal during task performance and MEG resting state low frequency activity.

Our results showing significant elevation in resting state low frequency activity in FEP is consistent with prior studies. Elevations in resting EEG/MEG theta band/low-alpha band activity in schizophrenia, including in FEP, have been consistently reported in the literature^4,5,46–51^.

The lack of differences in the magnitude of the 40 Hz MEG auditory evoked response (AER) between FEP and HC was somewhat surprising, because 40 Hz AER abnormalities have frequently been reported in medicated, chronic patients with schizophrenia [for review see ref. ^19,52^]. However, some studies have found minimal group differences on this measure^11^. A recent meta-analysis of 40-Hz AEP^19^ concluded that “the effect size of the auditory steady-state response deficit is currently lower than other electrophysiological indices of auditory dysfunction in schizophrenia.”

To our knowledge, only three EEG studies have reported data in FEP. Spencer^13^ enrolled 16 medicated FEP, Tada^20^ enrolled 13 medicated FEP, and Wang^21^ included 33 FEP, including 17 who were medication-naïve. All three studies reported alterations in the gamma band AER in FEP, although there were differences between those studies and ours which potentially could explain the different results. All three of these studies used EEG, and it has been shown that there can be differences in the cortical auditory evoked response between MEG and EEG^39^. In particular, these three studies performed their analysis at the scalp-surface sensor level, while our analysis of the auditory evoked responses was source-localized to auditory cortex. In addition, all three of these studies presented the auditory stimuli in block design with fixed interstimulus intervals, while this study presented the stimuli with stimulus conditions in shuffled order with a randomly varying interstimulus interval, to limit the effects of the previous history on each response.

It is possible that antipsychotic medication might have influenced the AEP; only two small studies have compared medication naïve subjects with treated patients^21,53^. It is possible that the lack of significantly reduced 40 Hz AEP response magnitude in FEP was due to a lack of statistical power in detecting a relatively weak effect with a small sample size, or perhaps the effect is less robust in FEP. Regardless, at least in this population and under these conditions, we did not find significantly less 40 Hz evoked response in FEP than HC. Our results strongly support the proposition that elevation in resting state low frequency brain activity is a more robust potential biomarker of early psychosis than abnormalities in the gamma band.

To our knowledge, we are the first to investigate the relationships between GABA levels and auditory evoked cortical gamma band oscillations in FEP subjects. In a combined group of chronic, medicated patients with schizophrenia and HC, Chen et al.^34^ reported a correlation between GABA levels and gamma oscillation measured with EEG at rest and during a working memory task. Here, we report no group difference in GABA levels, as well as a lack of correlation between GABA levels and auditory evoked gamma band oscillations in FEP or in HC. The lack of correlation is in agreement with the study of Wyss et al.^54^ carried out in healthy volunteers, which did not identify a correlation between GABA levels and auditory evoked gamma band response^54^ in a paradigm similar to the one used in our study. Others studies that have reported correlations between gamma activity and GABA levels in healthy volunteers were done using gamma oscillations generated in the visual^26^ and motor^27^ cortex. We note that MRS only gives the total tissue concentration of GABA, and tells nothing about its concentration in different tissue/cellular sub-compartments nor levels of neurotransmitter activity or turnover^55^. It is thus perhaps not that surprising that the link between MRS GABA and cortical gamma-band activity is not uniformly robust.

Several previous fMRI studies using the Stroop task have identified both behavioral and BOLD activation abnormalities in FEP relative to HC^56–58^. In order to test whether there were group differences in the relationship between the BOLD signal and resting state low frequency oscillations that are altered in FEP, we defined the neural circuit activated during the Stroop by pooling data from all participants and evaluated the correlations between activation in these regions and low frequency activity. Consistent with our prior analysis of these data, we observed increased BOLD activation in FEP compared to HC in several regions of the circuit^36^. In addition, in the combined group of FEP and HC, we found significant correlations between task-induced BOLD signal and low frequency activity; however, the correlation between these variables was not significant when considered for HC or FEP separately. These findings are in agreement with studies showing that theta, lower alpha, and upper alpha band coherence patterns are selectively correlated with task performance^59–62^.

While fMRI BOLD and MEG are very different modalities for exploring cortical function, studies have nevertheless found that the functional connectivity maps based on these measures can be similar^63–65^. Links between ongoing low-frequency EEG activity and task-related fMRI signals have also been found^66^. Given that low-frequency EEG band activity may be important for integrating cortical activity across long distances, and that the performance of a task requires the functional linking of disparate brain areas, the correlation between our low-frequency MEG resting state measures and the task-related fMRI Stroop is perhaps not that surprising.

Although the main purpose of this study was to mechanistically probe the relationships between different imaging modalities, this study allows us to compare the effect size of each modality that significantly distinguished FEP from HC. With a modest sample size, but taking advantage of the better signal to noise ratio and spectral resolution of a 7 T magnet and the better detectability of high-frequency auditory activity with MEG, we identified several measures that were significantly different between FEP and HC: altered low frequency oscillations (Cohen d (*d*) = 0.69), altered BOLD signal (*d* = 1.19), and as previously reported^35^, decreased tNAA (*d* = 0.77) and glutamate (*d* = 0.68) levels, findings that have now been replicated by another group at 7 T in FEP^32^.

A major limitation of this study is the modest sample size. We suggest that it could be important to replicate the results with a larger sample size. It was also a limitation that, while not exposed to long term chronic medication, nevertheless most of this population was being treated, with a variety of different medications and dosing. In the future, larger sample sizes may allow some of these factors to be regressed out. It must also be pointed out that even this study used only a fraction of current imaging modalities and cortical locations, which themselves are still limited by the scope of existing technology. In the long run the challenge will be to find the combination of imaging modalities that most efficiently parse the underlying heterogenous pathophysiology of SZ into a (hopefully) relatively few major ones, so that therapies may be more explicitly designed and targeted to the specific pathological mechanisms in individual patients. This study is just one step towards this goal.

In summary, in FEP, we report significant alterations in resting state low frequency activity, but no alterations in auditory evoked gamma band response, suggesting the former is a more robust biomarker of early psychosis. In addition, in this study, each of the three imaging modalities differentiated FEP from HC; fMRI with good effect size and MEG and MRS with moderate effect size.

## Methods

### Subjects

A total of forty nine subjects were recruited for this study. Twenty-three FEP patients determined by a clinician to be clinically stable were recruited from the University of Alabama at Birmingham’s outpatient psychiatric clinics. Twenty-six HC matched for age, gender, and family socio-economic status were recruited by advertisements in flyers and in the University’s newspaper. Exclusion criteria were major medical or neurological conditions, substance abuse within the past 6 months, previous serious head injury, history of loss of consciousness, and pregnancy. Consensus diagnoses were made according to DMS-V criteria in by two board certified psychiatrists from all historical and direct assessment information available (ACL and NVK), including the participants’ medical records The Brief Psychiatric Rating Scale “BPRS”^67^, and the Repeatable Battery for the Assessment of Neuropsychological Status “RBANS”^68^, were used to characterize symptom severity and general cognitive function. This study was approved by UABs Institutional Review Board, and all participants provided written informed consent.

Three FEP patients (long-term hospitalization, weight exceeding limit) and two HC (failed drug screen, MR contraindications) did not complete the MEG study, leaving 20 FEP and 24 HC for analyses. Two FEP (claustrophobia, weight-exceeding limit) and five HC (failed drug screen, MRI contraindication, lost to follow-up) did not complete the fMRI study. In addition, fMRI data were excluded for four FEP patients because of poor data quality, leaving 17 FEP patients and 21 HC for fMRI analyses. Two FEP (weight exceeding limit, claustrophobia) and five HC (failed drug screen, MR contraindications, lost to follow up) did not complete the MRS study, leaving 21 FEP and 21 HC for analyses.

FEP patients were treated with risperidone, with the following exceptions: one was unmedicated, two were treated with aripiprazole, one with clozapine, and one with a combination of zipradisone and clozapine. Additional demographic data is shown in Table 1.

**Table 1:** Demographics.

Measure	Control (HC, *n* = 24)	First episode psychosis (FEP, *n* = 22)	Statistic	*p*-value
Age, years	24.1 (5.0)	23.7 (4.7)	*t*(44) = 0.27	0.78
Sex, F/M	7/17	6/16	*χ*^2^(1) = 0.02	0.88
Smoker, yes/no	1/23	5/17	*χ*^2^(1) = 3.48	0.06
Parental SES^a^	3.4 (3.3)	3.6 (3.8)	*t*(43) = 0.10	0.91
RBANS^b^	93.8 (8.6)	74.0 (14.5)	*t*(39) = 5.39	<0.001
Illness duration, years^c^	–	1.02 (1.22)	–	–
BPRS^d^				
Total	–	32.8 (9.9)	–	–
Positive	–	7.1 (3.7)	–	–
Negative	–	5.9 (2.4)	–	–

Data are shown as Mean (SD).

^a^Socioeconomic Status. Information not available for one patient. Ranks determined from the Diagnostic Interview for Genetic Studies (1–18 scale); higher rank (lower numerical value) corresponds to higher socioeconomic status.

^b^Repeatable Battery for the Assessment of Neuropsychological Status. Information not available for three patients and two controls.

^c^Illness duration is in years from time of first treatment to time of first scan.

^d^Brief Psychiatric Rating Scale. Information not available for two FEP. Positive subscale: conceptual disorganization, hallucinatory behavior, and unusual thought content. Negative subscale: emotional withdrawal, motor retardation, and blunted affect.

### Magnetoencephalography (MEG)

A 4-D Systems Magnes WH (“Whole Head”) 148-channel magnetometer located in a shielded room below ground level were used for MEG recordings. Subjects lay on their backs. Fiducial head-sensor coils were used to ensure that the head had not moved during a scan. Additional head-sensor data were collected using a 3D digitizer and later used for registration between each subject’s MEG and anatomical MRI. Recordings were made at a sampling rate of 1 kHz, with a bandpass filter from 0.1 to 200 Hz.

Each subject completed two separate scanning runs, of 5 min each, in the following order.

1. Resting state. Subjects rested quietly with their eyes closed. These data have been previously analyzed in another study for another purpose^37^.
2. Preattentive audio. Subjects rested quietly with their eyes closed, and listened to a series of acoustic tones delivered through plastic tubes into each ear. The tones were either a 500 ms duration burst of 40 Hz clicks, a 500 ms duration burst of 20 Hz clicks, or 500 ms of silence. The tones were delivered in pseudo-random shuffled order, with inter-trial intervals varying randomly from 500 to 750 ms. There were approximately 80 trials of each type per scanning run for each subject. Here we only present the data for the 40 Hz tones.

Visual inspection of the real-time display showed no MEG correlates of sleep. Additionally, all of the subjects were instantly responsive upon completion of each 5-min block and verbally responsive to instructions.

### Functional task

Participants completed a computerized version of the Stroop color-naming task during the scan^69^. An IFIS-SA system utilizing E-Prime was used to present task stimuli to participants and record their response and response time. For each trial, participants were presented one of three words, “RED”, “GREEN”, or “BLUE,” and the color of the font was one of these same three colors. Participants were instructed to ignore the lexical meaning of the word and instead indicate the color of the font by pressing one of three buttons. Congruent trials were trials where the lexical meaning matched the font color, and incongruent trials were trials where it did not. Participants completed two sessions consisting of sixty-four trials each (30% incongruent).

### Image acquisition/preprocessing

Imaging was performed on a whole body MAGNETOM 7 T MRI scanner (Siemens Healthineers, Erlangen Germany) equipped with a 32-channel head coil (Nova Medical) at the Auburn University MRI Research Center. A structural scan was acquired for anatomical reference (MPRAGE; TR/TE/TI 2200/2.96/1050 ms, flip angle = 7°, GRAPPA acceleration factor = 2, FOV = 224 × 224 mm, 0.7 mm isotropic voxels).

The anatomical scan was used to guide spectroscopy voxel placement in the bilateral dorsal (ACC) (2.7 × 2 × 1 cm^3^). After shimming with FASTESTMAP (fast, automatic shim technique using echo-planar signal readout for mapping along projections) and optimization of the radiofrequency power, spectra were acquired using an ultrashort echo time stimulated echo acquisition mode (STEAM) sequence (TR/TE/TM = 10,000/5/45 ms, 32 averages, 4 kHz bandwidth, 2048 points), outer volume suppression, and VAPOR (variable power RF pulses and optimized relaxation delays) water suppression. Two averages of unsuppressed water scans were obtained as reference. During the MRS scan participants were instructed to keep their eyes open. Spectra were processed in LCModel (version 6.3-1) using a simulated basis set and default processing parameters [also see ref. ^45^]. Metabolite levels were corrected for partial volume using Gasparovic et al.’s method^70^. The analysis of these data has been previously published^45^.

Task fMRI data were acquired using the gradient recalled echo-planar (EPI) sequence (TR/TE = 3000/28, flip angle = 70°, FOV = 200 × 200 mm, voxel size = 0.85 × 0.85 × 1.8 mm, 1 mm gap, 37 axial slices, 120 acquisitions per session). A second anatomical scan was acquired for co-registration of the functional images (TR/TE/TI 2000/2.89/1050 ms, flip angle = 7°, GRAPPA acceleration factor = 2, FOV = 190 × 190 mm, 0.7 mm isotropic voxels). Data analyses were performed in SPM12. Preprocessing included realigning, unwarping, co-registering to the MPRAGE, normalizing to MNI space, and smoothing with a 5 mm full width at half maximum (FWHM) Gaussian kernel. A single-subject voxel-by-voxel whole-brain general linear model was calculated for each individual. Five conditions were included in the event related model: correct nonrepeat incongruent trials, correct nonrepeat congruent trials, error trials, no response trials, and repeat trials [also see ref. ^58^]. Repeat trials were trials, where both the lexical meaning and font color of the word were identical to the previous trial. Motion parameters were used as nuisance regressors. Some data from this fMRI study have been reported previously^36^. For this study, we defined the neural circuit activated in the Incongruent versus Congruent trials by pooling data from all participants (FEP and HC). The significance was assessed using voxel (*p*_uncorrected_ < 0.01) and cluster level correction (*p*_FDR_ < 0.01).

### MEG analysis

MEG data were analyzed using the “Brainstorm” software package^71^. Pre-processing of the raw data included the removal of line noise using a notch filter (at 60, 120, 180, 240, and 300 Hz) and high-pass filtering at 0.3 Hz. Cardiac and motion artifacts were removed using an independent component analysis. Additional artifacts were removed using a signal-space projection method.

Analysis of the resting state data was done in sensor space. Analysis at the different frequency bands was performed by dividing up the entire 5 min duration recording period into 2 s epochs, applying a Hamming window, and then averaging the magnitude of the Fourier transform over all epochs for each magnetometer channel separately.

Analysis of the auditory evoked responses was done in brain space. The data for each individual subject was registered to high-resolution structural MRI scans. Prior to MEG-fMRI co-registration, cortical surfaces were extracted using the Freesurfer image analysis suite (http://surfer.nmr.mgh.harvard.edu/) and inspected using Freesurfer’s Quality Assurance tools. Using Minimum Norm imaging (MNE), a single time series was extracted from the left and right auditory cortices for each subject. Three analysis were performed on these time series, separately for the left and right auditory cortices:

1. Root mean square (RMS) power of the mean time-locked evoked response bandpass filtered to 35–45 Hz for the 40 Hz tones.
2. The event-related spectral perturbation (ERSP). This measures changes in signal power that is induced by, but not necessarily phase-locked to, the appearance of the stimulus^21^. For each trial, the RMS value of the cortical signal from 0 to −100 ms before stimulus onset, was subtracted from the RMS value of the cortical signal from 0 to 500 ms after stimulus onset, and these values averaged across all trials. The signals were first bandpass filtered to 35–45 Hz for the 40 Hz tones.
3. The phase locking factor. Sometimes also referred to as the Intertrial Coherence, this quantifies the degree to which the evoked response is phase-locked to the phase of the auditory stimulus^21^. A value of 0 indicates no relationship between stimulus and response phases, and a value of 1 indicates a perfect trial-by-trial relationship between the stimulus phase and the evoked cortical signal phase. It was calculated as the circular variance of the phases. The signals were first bandpass filtered to 35–45 Hz for the 40 Hz tones.

### Statistical analyses

We had two very specific a priori hypotheses based on the existing literature, as outlined in the introduction: 1. For SZ compared to HC, increased low frequency MEG fluctuations during rest, and altered MEG gamma-band oscillations in response to gamma-band auditory stimuli, and: 2. A relationship between gamma frequency and MRS GABA in controls that is disrupted in patients. We tested these hypotheses using *t*-tests (two-tailed) and linear regression (Pearson’s correlation coefficient) using MATLAB R2019a. We set significance as *p* < 0.05. For the rest of the data we conducted exploratory tests, again using *t*-tests and linear regression with significance set at *p* < 0.05. We did not calculate interaction terms, as this requires upwards of four times as much data to detect an effect^72^, and our sample sizes here were too small for that.

### Reporting summary

Further information on experimental design is available in the Nature Research Reporting Summary linked to this paper.

## Supplementary information

Supplemental Material

Reporting Summary Checklist FLAT

## Data Availability

The data that support the findings of this study are available from the corresponding author upon reasonable request.

## Code availability

The code used to analyze the data in this study is available from the corresponding author upon reasonable request.
