# Supplementary material for: A multimodal magnetoencephalography 7 T fMRI and 7 T proton MR spectroscopy study in first episode psychosis
Source: NPJ Schizophr. 2020 Sep 4;6:23. doi: 10.1038/s41537-020-00113-4 (PMC7473853; doi:10.1038/s41537-020-00113-4)
Supplement: Supplementary file 1 — Supplemental Material [file 41537_2020_113_MOESM1_ESM.pdf]

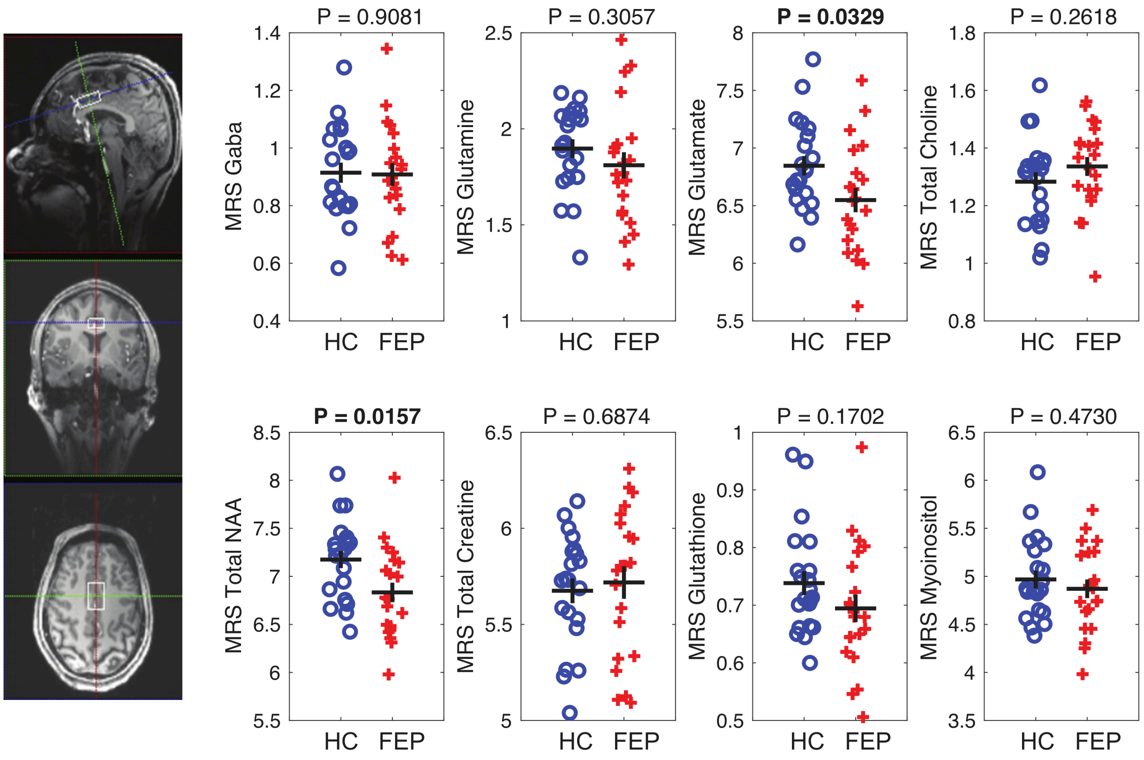

Supplementary Figure 1. Magnetic Resonance Spectroscopy results. Left: placement of voxel in the bilateral anterior cingulate cortex (ACC) in one example subject. Right: Values of neurometabolites as determined from 7T MRS spectroscopy from the ACC for both HC (open blue circles) and FEP (red plus signs). Individual symbols are for individual subjects. The horizontal black bars are the means for either FEP or HC for each neurometabolite, and the vertical black bars represent +/- one standard error of the mean (SEM). The values for total NAA and Glutamate were different for HC and FEP by t-test (*p*< 0.05). Data are replotted from (35).

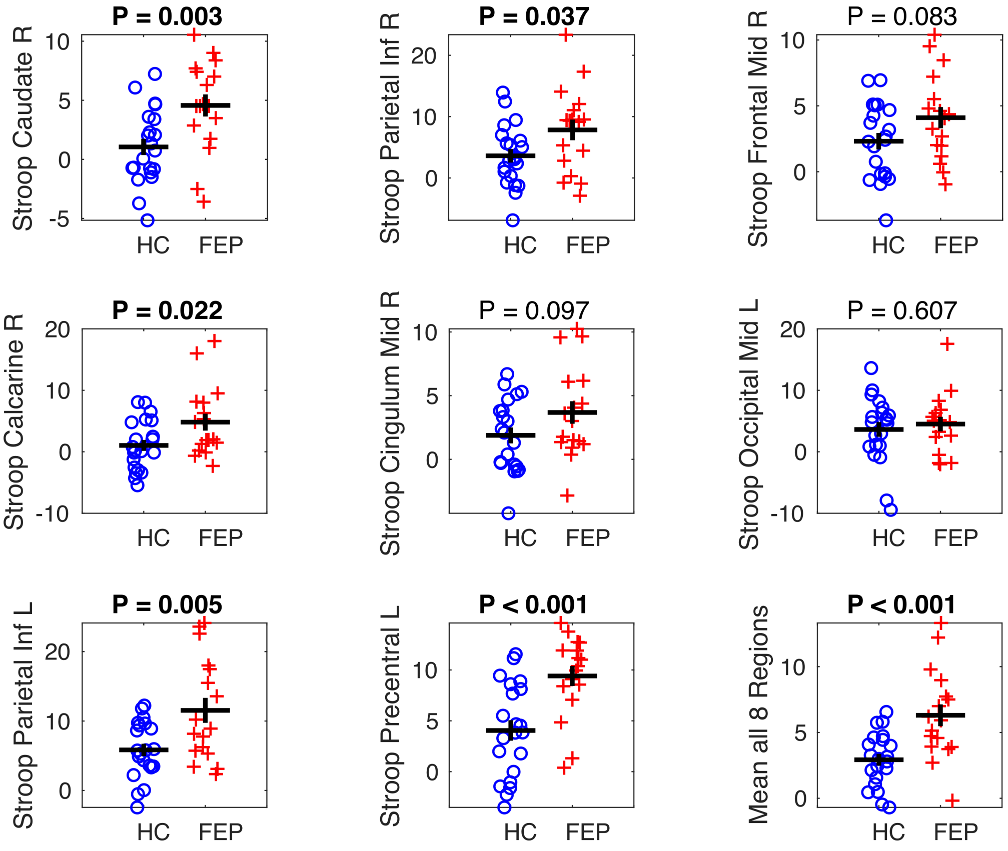

Supplementary Figure 2. Plot of the Stroop effect fMRI signal Incongruent > Congruent for both HC (blue circles) and FEP (red crosses) for the eight different brain regions listed in Supplementary Table 1, and for the mean of all eight regions (lower right sub-panel). The significance level of the T-test comparing HC and FEP is given above each plot.

Caudate R:	P = 0.003,	t = - 3.085,	df = 36
Parietal Inf R:	P = 0.037,	t = -2.169,	df = 36
Frontal Mid R:	P = 0.083,	t = -1.786,	df = 36
Calcarine R:	P = 0.022,	t = -2.389,	df = 36
Cingulum Md R:	P = 0.097,	t = -1.705,	df = 36
Occipital Mid L:	P = 0.607,	t = -0.518,	df = 36
Parietal Inf L:	P = 0.005,	t = 3.009,	df = 36
Precentral L:	P < 0.001,	t = -3.758,	df = 36
Mean 8 Regions:	P < 0.001,	t = -3.775,	df = 36.

Supplementary Table 1. Regions of significant difference in fMRI signal for the Incongruent > Congruent trials of the Stroop task.

Montreal Neurological Institute Coordinates (X, Y, Z)	Region Name
1. 13, 0, 16	Caudate_R
2. 28, -64, 45	Parietal_Inf_R
3. 35, 25, 1	Frontal_Mid_R
4. 39, -82, 7	Calcarine_R
5. 4, -24, 28	Cingulum_Mid_R
6. -22, -96, 9	Occipital_Mid_L
7. -30, -58, 48	Parietal_Inf_L
8. -46, 19, 28	Precentral_L
