# Supplementary material for: A multimodal magnetoencephalography 7 T fMRI and 7 T proton MR spectroscopy study in first episode psychosis
Source: NPJ Schizophr. 2020 Sep 4;6:23. doi: 10.1038/s41537-020-00113-4 (PMC7473853; doi:10.1038/s41537-020-00113-4)
Supplement: Supplementary file 2 — Reporting Summary Checklist FLAT [file 41537_2020_113_MOESM2_ESM.pdf]

nature research

Corresponding author(s): Adrienne C. Lahti

Last updated by author(s): May 17, 2020

Reporting Summary

Nature Research wishes to improve the reproducibility of the work that we publish. This form provides structure for consistency and transparency in reporting. For further information on Nature Research policies, see our Editorial Policies and the Editorial Policy Checklist.

Statistics

For all statistical analyses, confirm that the following items are present in the figure legend, table legend, main text, or Methods section.

n/a	Confirmed	
☐	☒	The exact sample size (*n*) for each experimental group/condition, given as a discrete number and unit of measurement
☐	☒	A statement on whether measurements were taken from distinct samples or whether the same sample was measured repeatedly
☐	☒	The statistical test(s) used AND whether they are one- or two-sided<br>*Only common tests should be described solely by name; describe more complex techniques in the Methods section.*
☒	☐	A description of all covariates tested
☒	☐	A description of any assumptions or corrections, such as tests of normality and adjustment for multiple comparisons
☐	☒	A full description of the statistical parameters including central tendency (e.g. means) or other basic estimates (e.g. regression coefficient) AND variation (e.g. standard deviation) or associated estimates of uncertainty (e.g. confidence intervals)
☐	☒	For null hypothesis testing, the test statistic (e.g. *F*, *t*, *r*) with confidence intervals, effect sizes, degrees of freedom and *P* value noted<br>*Give P values as exact values whenever suitable.*
☒	☐	For Bayesian analysis, information on the choice of priors and Markov chain Monte Carlo settings
☒	☐	For hierarchical and complex designs, identification of the appropriate level for tests and full reporting of outcomes
☐	☒	Estimates of effect sizes (e.g. Cohen's *d*, Pearson's *r*), indicating how they were calculated

Our web collection on statistics for biologists contains articles on many of the points above.

Software and code

Policy information about availability of computer code

Data collection	Data were collected using the standard commercial inbuilt software supplied with the MAGNETOM 7T MRI scanner (Siemens Healthineers, Erlangen Germany) and the 4-D Systems Magnes WH ("Whole Head") 148-channel magnetometer
Data analysis	As described in the manuscript, data were analyzed using Brainstorm, SPSS, LCMOdel 6.3-1, and Matlab R2019a

For manuscripts utilizing custom algorithms or software that are central to the research but not yet described in published literature, software must be made available to editors and reviewers. We strongly encourage code deposition in a community repository (e.g. GitHub). See the Nature Research guidelines for submitting code & software for further information.

Data

Policy information about availability of data

All manuscripts must include a data availability statement. This statement should provide the following information, where applicable:

- Accession codes, unique identifiers, or web links for publicly available datasets
- A list of figures that have associated raw data
- A description of any restrictions on data availability

Data Availability
The data that support the findings of this study are available from the corresponding author upon reasonable request.

Field-specific reporting

Please select the one below that is the best fit for your research. If you are not sure, read the appropriate sections before making your selection.

☒ Life sciences ☐ Behavioural & social sciences ☐ Ecological, evolutionary & environmental sciences

For a reference copy of the document with all sections, see nature.com/documents/nr-reporting-summary-flat.pdf

Life sciences study design

All studies must disclose on these points even when the disclosure is negative.

Sample size	Sample size was determined by using previous experience with similar studies on a similar population.
Data exclusions	From the methods section: Three FEP patients (long-term hospitalization, weight exceeding limit) and two HC (failed drug screen, MR contraindications) did not complete the MEG study, leaving 20 FEP and 24 HC for analyses. Two FEP (claustrophobia, weight-exceeding limit) and five HC (failed drug screen, MRI contraindication, lost to follow-up) did not complete the fMRI study. In addition, fMRI data were excluded for four FEP patients because of poor data quality, leaving 17 FEP patients and 21 HC for fMRI analyses. Two FEP (weight exceeding limit, claustrophobia) and five HC (failed drug screen, MR contraindications, lost to follow up) did not complete the MRS study, leaving 21 FEP and 21 HC for analyses.
Replication	We compared our results with the published literature. We replicated previously published effects of psychosis on MRS glutamate and tNAA, MEG low-frequency resting state, and fMRI task-related functional activation. We did not replicate previous findings of a deficit in the MEG auditory evoked 40 Hz response, but we have extensively commented on this in the discussion.
Randomization	We recruited one first episode psychosis patient group. We recruited healthy controls to match the demographics of this group.
Blinding	No blinding was performed. It was not possible to do this during data collection as it was obvious if the people being scanned were patients or healthy controls. Blinding was not deemed necessary for the analysis as none of the analysis methods required individual judgment.

Reporting for specific materials, systems and methods

We require information from authors about some types of materials, experimental systems and methods used in many studies. Here, indicate whether each material, system or method listed is relevant to your study. If you are not sure if a list item applies to your research, read the appropriate section before selecting a response.

Materials & experimental systems

n/a	Involved in the study	
☒	☐	Antibodies
☒	☐	Eukaryotic cell lines
☒	☐	Palaeontology and archaeology
☒	☐	Animals and other organisms
☐	☒	Human research participants
☒	☐	Clinical data
☒	☐	Dual use research of concern

Methods

n/a	Involved in the study	
☒	☐	ChIP-seq
☒	☐	Flow cytometry
☐	☒	MRI-based neuroimaging

Human research participants

Policy information about studies involving human research participants

Population characteristics	(see table 1 in manuscript for more detail). Control: age mean 24.1 (5.0 SD), sex F/M 7/17, Parental SES mean 3.4 (3.3 SD), RBANS 93.8 (8.6 SD). Patients: age mean 23.7 (4.7 SD), sex F/M 6/16, Parental SES mean 3.6 (3.8 SD), RBANS mean 74.0 (14.5 SD).
Recruitment	(From methods section in manuscript) A total of forty nine subjects were recruited for this study. Twenty-three FEP patients determined by a clinician to be clinically stable were recruited from the University of Alabama at Birmingham's outpatient psychiatric clinics. Twenty-six HC matched for age, gender, and family socio-economic status were recruited by advertisements in flyers and in the University's newspaper. Exclusion criteria were major medical or neurological conditions, substance abuse within the past six months, previous serious head injury, history of loss of consciousness, and pregnancy. Consensus diagnoses were made according to DMS-V criteria in by two board certified psychiatrists from all historical and direct assessment information available (ACL and NVK), including the participants' medical records The Brief Psychiatric Rating Scale ("BPRS," 44) and the Repeatable Battery for the Assessment of Neuropsychological Status ("RBANS," 45) were used to characterize symptom severity and general cognitive function.
Ethics oversight	Institutional Review Board (IRB of the University of Alabama at Birmingham (UAB)

Note that full information on the approval of the study protocol must also be provided in the manuscript.

Magnetic resonance imaging

Experimental design

Design type: MR Spectroscopy resting state; fMRI Stroop tesk

Design specifications: *Specify the number of blocks, trials or experimental units per session and/or subject, and specify the length of each trial or block (if trials are blocked) and interval between trials.*

Behavioral performance measures: *State number and/or type of variables recorded (e.g. correct button press, response time) and what statistics were used to establish that the subjects were performing the task as expected (e.g. mean, range, and/or standard deviation across subjects).*

Acquisition

Imaging type(s): MR Spectroscopy resting state; fMRI Stroop tesk

Field strength: 7 T

Sequence & imaging parameters: Imaging was performed on a whole body MAGNETOM 7T MRI scanner (Siemens Healthineers, Erlangen Germany) equipped with a 32-channel head coil (Nova Medical) at the Auburn University MRI Research Center. A structural scan was acquired for anatomical reference (MPRAGE; TR/TE/TI 2200/2.96/1050msec, flip angle= 7°, GRAPPA acceleration factor= 2, FOV= 224 x 224mm, 0.7mm isotropic voxels).
The anatomical scan was used to guide spectroscopy voxel placement in the bilateral dorsal (ACC) (2.7× 2× 1cm3). After shimming with FASTESTMAP (fast, automatic shim technique using echo-planar signal readout for mapping along projections) and optimization of the radiofrequency power, spectra were acquired using an ultra-short echo time stimulated echo acquisition mode (STEAM) sequence (TR/TE/TM= 10,000/5/45msec, 32 averages, 4 kHz bandwidth, 2048 points), outer volume suppression, and VAPOR (variable power RF pulses and optimized relaxation delays) water suppression. Two averages of unsuppressed water scans were obtained as reference. During the MRS scan participants were instructed to keep their eyes open. Spectra were processed in LCModel (version 6.3-1) using a simulated basis set and default processing parameters [also see (47)]. Metabolite levels were corrected for partial volume using Gasparovic et al.'s method (48). The analysis of these data has been previously published (47)
Task fMRI data were acquired using the gradient recalled echo-planar (EPI) sequence (TR/TE= 3000/28, flip angle= 70°, FOV= 200x 200mm, voxel size= 0.85x 0.85x 1.8mm, 1mm gap, 37 axial slices, 120 acquisitions per session). A second anatomical scan was acquired for co-registration of the functional images (TR/TE/TI 2000/2.89/1050msec, flip angle= 7°, GRAPPA acceleration factor= 2, FOV= 190 X 190mm, 0.7mm isotropic voxels).

Area of acquisition: Anterior Cingulate cortex for MRS Spectroscopy; whole brain for task based

Diffusion MRI: ☐ Used ☒ Not used

Preprocessing

Preprocessing software: Data analyses were performed in SPM12. Preprocessing included realigning, unwarping, co-registering to the MPRAGE, normalizing to MNI space, and smoothing with a 5mm full width at half maximum (FWHM) Gaussian kernel.

Normalization: Normalized to MNI space

Normalization template: MNI

Noise and artifact removal: Motion parameters were used as nuisance regressors.

Volume censoring: Volume censoring not used.

Statistical modeling & inference

Model type and settings: A single-subject voxel-by-voxel whole-brain general linear model was calculated for each individual. Five conditions were included in the event related model: correct non-repeat incongruent trials, correct non-repeat congruent trials, error trials, no response trials, and repeat trials [also see (49)]. Repeat trials were trials where both the lexical meaning and font color of the word were identical to the previous trial.

Effect(s) tested: For the task study, we defined the neural circuit activated in the Incongruent versus Congruent trials by pooling data from all participants (FEP and HC). The significant was assessed using voxel (puncorrected < 0.01) and cluster level correction (pFDR < 0.01).

Specify type of analysis: ☐ Whole brain ☐ ROI-based ☒ Both

Anatomical location(s): Task-specific identified ROIs as identified in the analysis for the functional study.

Statistic type for inference (See Eklund et al. 2016)	For this study, we defined the neural circuit activated in the Incongruent versus Congruent trials by pooling data from all participants (FEP and HC). The significant was assessed using voxel (puncorrected < 0.01) and cluster level correction (pFDR < 0.01).
Correction	Cluster level correction pFDR < 0.01.

Models & analysis

n/a	Involved in the study
☒	☐ Functional and/or effective connectivity
☒	☐ Graph analysis
☒	☐ Multivariate modeling or predictive analysis
